# Recombinant IL-37 Exerts an Anti-inflammatory Effect on Human Aortic Valve Interstitial Cells through Extracellular and Intracellular Actions

**DOI:** 10.7150/ijbs.85745

**Published:** 2023-07-31

**Authors:** Erlinda The, Yufeng Zhai, Qingzhou Yao, Lihua Ao, Suzhao Li, David A. Fullerton, Charles A. Dinarello, Xianzhong Meng

**Affiliations:** Departments of Surgery and Medicine, University of Colorado Denver, Aurora, CO 80045.

**Keywords:** aortic valve, endocytic pathway, IL-37, inflammation, nuclear action

## Abstract

Calcific aortic valve disease (CAVD) is a chronic inflammatory disease with slow progression that involves soluble extracellular matrix (ECM) proteins. Previously, we found that recombinant interleukin (IL)-37 suppresses aortic valve interstitial cells (AVIC) inflammatory response through the interaction with IL-18 receptor α-chain (IL-18Rα) on the cell surface. Endogenous IL-37 can be retained in the cytoplasm or released into extracellular spaces. It remains unknown whether recombinant IL-37 exerts the anti-inflammatory effect through intracellular action. Here, we found that recombinant IL-37 suppressed AVIC inflammatory response to soluble ECM proteins. Interestingly, recombinant IL-37 was internalized by human AVICs in an IL-18Rα-independent fashion. Blocking endocytic pathways reduced the internalization and anti-inflammatory potency of recombinant IL-37. Overexpression of IL-37 in human AVICs suppressed soluble ECM proteins-induced NF-κB activation and the production of ICAM-1 and VCAM-1. However, IL-37D20A (mutant IL-37 lacking nucleus-targeting sequences) overexpression had no such effect, and the inflammatory response to soluble ECM proteins was essentially intact in AVICs from transgenic mice expressing IL-37D20A. Together, recombinant IL-37 can be internalized by human AVICs through endocytosis. Intracellular IL-37 exerts an anti-inflammatory effect through a nucleus-targeting mechanism. This study highlights the potent anti-inflammatory effect of recombinant IL-37 in both extracellular and intracellular compartments through distinct mechanisms.

## Introduction

Calcific aortic valve disease (CAVD) is the most common valvular heart disease in the elderly 1, and it accounts for approximately 15,000 deaths annually in United States 2. The aging American population has resulted in a dramatic rise in the prevalence of CAVD. Clinical treatment for CAVD is currently limited to surgical or transcatheter aortic valve replacement, however, these procedures involve significant risk 3. The lack of pharmacological intervention to prevent CAVD progression is a major challenge in the management of this disease 4.

Chronic inflammation is recognized as a hallmark of CAVD 4. Inflammatory mediators play an important role in initiating and sustaining progressive fibrotic tissue remodeling, leading to valvular calcification in CAVD 5. Aortic valve interstitial cells (AVICs) are the dominant cellular components of aortic valve leaflets that maintain valvular structure and function via regulation of valve extracellular matrix (ECM) remodeling 6. The pathogenesis of CAVD arises largely due to AVIC inflammatory activity, mediated by pathogen-associated molecular patterns or damage-associated molecular patterns (DAMPs) 7. Mounting evidence demonstrates that soluble ECM proteins can function as DAMPs and promote valvular inflammation, fibrosis, and calcification 8-11. Studies have found correlations between abnormal levels of ECM proteins and aortic valve lesions 12. Matrilin-2 and biglycan are ECM proteins expressed in most tissues. They are released from the ECM and become soluble in response to tissue injury or stress 13, 14. The expression of both matrilin-2 and biglycan is elevated in the condition of inflammatory diseases 13, 15. We have reported that soluble matrilin-2 and biglycan elevate inflammatory and osteogenic activities in human AVICs by activating Toll-like receptors (TLR) 2 and 4 8-11, 16.

Interleukin (IL)-37, a unique member of the IL-1 family, functions as a natural suppressor of innate and acquired immunity 17, 18. IL-37 is expressed in human tissue, including aortic valve, but not found in mice 4, 19. Lower levels of IL-37 are associated with chronic inflammatory diseases 20, 21. IL-37 limits inflammation by reducing the production of pro-inflammatory cytokines and blocking the differentiation and activation of macrophages 22, 23. Knockdown of IL‑37 in human renal tubular epithelial cells and primary peripheral blood mononuclear cells increase the production of IL-1β, tumor necrosis factor (TNF)-α and IL-6 induced by inflammatory stimuli and cytokines 24. Additionally, the expression of human IL-37 in transgenic mice markedly reduces the production of pro-inflammatory cytokines and chemokines following TLR4 stimulation 25, 26. Our recent work demonstrated that IL-37 suppresses inflammatory response of AVIC to TLR2/4 stimulation, which alleviates aortic valve lesions in mice exposed to a pro-inflammatory stimulus or high fat diet 4, 27.

Endogenous IL-37 can be secreted into the extracellular spaces or retained in the cytoplasm 28. Previous studies, including ours, found that extracellular IL-37 exerts its anti-inflammatory effect mainly through interaction with IL-18 receptor α-chain (IL-18Rα) 27, 29. Intracellular IL-37 undergoes maturation by caspase-1 processing and subsequently translocates into the nucleus. This causes downregulation of pro-inflammatory cytokines 25, 26. It remains unclear if recombinant IL-37 exerts its anti-inflammatory effect through intracellular action.

In the current study, we aimed: 1) to study whether human AVICs uptake recombinant IL-37; 2) to determine whether internalized recombinant IL-37 exerts its anti-inflammatory effect intracellularly in human AVIC; and 3) to identify the action site of intracellular IL-37.

## Materials and Methods

### Chemicals and Reagents

Recombinant human proteins IL-37, matrilin-2 (endotoxin free), biglycan (endotoxin free), as well as neutralizing antibody against IL-18Rα were purchased from R&D Systems (Minneapolis, MN). Protein tagged Alexa FluorTM 555 microscale protein labeling kit and medium 199 were purchased from Thermo Fisher Scientific (Waltham, MA). Nystatin, chlorpromazine, amiloride, lipopolysaccharide (LPS, Escherichia coli 0111:B4), Tween 20, gelatin, collagenase and other reagents were purchased from Sigma-Aldrich (St. Louis, MO). Antibody against intercellular adhesion molecule 1 (ICAM-1) was purchased from Santa Cruz Biotechnology (Santa Cruz, CA). Antibodies against phospho-double-stranded RNA-dependent protein kinase (PKR) T446, total PKR, histone H3 and β-actin were obtained from Abcam (Cambridge, MA). Antibodies against phosphor-nuclear factor kappa-light-chain-enhancer of activated B cells (NF-κB) p65, total NF-κB p65, vascular cell adhesion protein 1 (VCAM-1), early endosome antigen (EEA)1 and Ras-related protein (Rab)7 were purchased from Cell Signaling Technology (Beverly, MA).

### Isolation and Culture of Human AVICs

Normal tricuspid aortic valves with no abnormalities were collected at the University of Colorado Hospital from explanted hearts of heart-transplant patients with cardiomyopathy. This study was performed in accordance with the Declaration of Helsinki 1964 and revision 2013 and was approved by the Institutional Review Board of University of Colorado. Written, informed consent was obtained from all aortic valve donors.

Normal human aortic valve leaflets were washed in 1X phosphate-buffered saline (PBS, Thermo Fisher Scientific, Waltham, MA), cut into small pieces, and digested in a collagenase solution (type I, 1 mg/mL) at 37℃ for 30 minutes to remove endothelial cells. The remaining tissue was treated with a fresh solution of 1 mg/mL collagenase by stirring the digestion solution at 37 ℃ over 4-6 hours to break up the tissue mass. AVIC suspension was centrifuged at 1,000 rpm for 10 minutes. The resulting cell pellets were resuspended and cultured in M199 medium and supplemented with 10% fetal bovine serum (FBS), 100 U/mL of Penicillin, and 100 µg/mL of Streptomycin at 37℃ in a humidified incubator with 5% CO2. The medium was replaced every 3 days throughout the growth and experimental periods. Cells of passage 3 to 6 at 90% confluence were used for this study. All experiments in this study were repeated using AVICs isolated from five different donor valves.

AVICs from aortic valve of wild type (WT), IL-37 transgenic (IL-37tg), and mutated D to A site in IL-37b (IL-37D20A, to prevent translocation of IL-37 to the nucleus) mice were isolated as described previously 4, 30. Briefly, aortic valve tissues were washed in PBS and digested with collagenase type I (1 mg/mL). To initiate AVIC outgrowth, aortic valve tissue fragments were plated on the 1% gelatin-coated dishes and cultured with medium 199 containing 10% FBS, 100 U/mL of Penicillin, and 100µg/mL of Streptomycin. The cells were then collected and passaged. Cells were treated with or without recombinant IL-37 (1 ng/mL) prior to treatment with recombinant matrilin-2 (2 μg/mL) 11 or biglycan (0.2 μg/mL) 9 for 48 hours. In a previous study, we demonstrated that a concentration of 1 ng/mL of recombinant IL-37 effectively suppresses the inflammatory and osteogenic activities induced by oxidized low density lipoprotein and pro-inflammatory stimuli in human AVICs 4. Cell lysate was used for the assessment of protein levels of PKR, NF-κB p65, ICAM-1 and VCAM-1.

### Plasmid and Cell Transfection

For transfection of human AVICs, full length IL-37b or IL-37D20A was released from plasmid pTarget and inserted into a pIRES vector with an enhanced green fluorescent protein (EGFP) expression sequence. Both plasmids were developed by Charles Dinarello's laboratory. Human AVICs were transfected with plasmids encoding IL-37 and IL-37D20A using Lipofectamine 3,000 (Thermo Fisher Scientific, Waltham, MA) according to the manufacturer's instructions. After 7 days of treatment, cells were collected for assessment of the effects of IL-37 and IL-37D20A overexpression on pro-inflammatory signaling and the protein expression of cell adhesion molecules.

### Generation of IL-37tg and IL-37D20Atg Mice

The IL-37tg and IL- 37D20Atg mice were described in the previous studies 22, 31. Fertilized eggs from C57BL/6 mice were injected with the pIRES-EGFP-IL-37b or pIRES-EGFP-IL-37bD20A expression plasmid and implanted into C57BL/6 females 22. The animal experiments were approved by University of Colorado Denver Animal Care and Use Committee, and this investigation conforms to the Guide for the Care and Use of Laboratory Animals (National Research Council, revised 1996).

### Preparation of Alexa 555-labeled IL-37

To investigate the internalization of recombinant IL-37 into human AVICs and co-localization with other proteins in cells, IL-37 was labeled using the Alexa Fluor 555 Protein Labeling Kit (Thermo Fisher Scientific, Waltham, MA)) according to instructions provided by the manufacturer. In brief, 1 M sodium bicarbonate solution (pH 8.3) were added to 1 mg/mL solution of recombinant IL-37, followed by incubation with the reactive dye in the vial for 15 minutes at room temperature. To remove excess dye, the reaction mixture was added into a spin column contain resin. The final Alexa 555-labeled IL-37 was stored at -20 °C.

### Immunoblotting

Immunoblotting was applied to analyze ICAM-1, VCAM-1, phospho- and total-NF-κB, phospho- and total-PKR, IL-37 and histone H3. Cells were lysed in 2x Laemmli sample buffer (Biorad, Hercules, CA). To assess intracellular of IL-37 in AVICs, nuclear and cytoplasmic extractions were performed by using CelLyticTM NuCLEARTM extraction kit (Sigma-Aldrich, St. Louis, MO) following the manufacturer's instruction. All samples were run on 4-20% SDS-PAGE gels and transferred to nitrocellulose membranes. The membranes were incubated with 5% skim milk solution for 1 hour at room temperature, followed with primary antibodies solution (1:200 to 1:1000 volume dilution) overnight at 4℃. The secondary antibodies were applied at a concentration of 1:10,000 and the immunoblots were visualized using enhanced chemiluminescence. β-actin was used to assess protein loading. In phosphorylation assay, total NF-κB and total PKR were used for normalization. Western blots were quantified by densitometry using the ImageLab software of the Bio-Rad.

### Immunofluorescence Staining

Cells were treated with or without neutralizing antibody against IL-18Rα prior to the application of Alexa 555-tagged IL-37. The cells were permeabilized with methanol/acetone (70/30 v/v) solution and fixed with 4% paraformaldehyde for 15 minutes, followed by 30 minutes of incubation with 10% goat serum at room temperature to block non-specific binding. Some cells were then stained overnight at 4 °C with primary antibodies against EEA1 (1:100, biomarker for early endosomes) and Rab7 (1:100, biomarker for late endosomes) to identify whether IL-37 was internalized into endosomes. Cells were washed and incubated with secondary antibodies that were conjugated to Alexa Fluor 488 (1:150) for 2 hours at room temperature. The nucleus was identified using 4′,6-Diamidino-2-phenylindole (DAPI) staining, while wheat germ agglutinin (WGA) was utilized to visualize the cell surface. Images were captured using a Leica CTR5500 digital microscope (Leica Mikroskopie und Systeme GmbH, Wetzlar, Germany).

### ELISA

The levels of C-C motif chemokine ligand 2 (CCL2) and IL-6 in cell culture supernatants were measured using commercial ELISA kits (R & D Systems, Minneapolis, MN) according to the manufacturer's instructions. Samples and standards were measured in triplicate with an automatic microplate reader (Biotek, Winooski, VT) at a wavelength of 450 nm.

### Statistical Analysis

Data are presented as mean ± standard error of the mean (SEM). Significance of differences was evaluated with an unpaired Student's t-test or one-way ANOVA with Tukey's multiple comparisons test. A *P* value ≤ 0.05 was considered to be statistical significant. Statistical analysis was performed using GraphPad Prism 9 (GraphPad Software, San Diego, CA).

## Results

### Recombinant IL-37 suppresses AVIC inflammatory response induced by soluble ECM proteins

To evaluate the pro-inflammatory effect of soluble ECM proteins on AVICs, we treated human AVICs with different doses of recombinant matrilin-2 (0.5-2 μg/mL) or biglycan (0.05-0.2 μg/mL) for 48 hours. As shown in **Figure S1**, recombinant matrilin-2 markedly up-regulated the level of adhesion molecule ICAM-1 at 2 μg/mL, while recombinant biglycan increased ICAM-1 levels at 0.2 μg/mL. This suggests that biglycan is more potent than matrilin-2 in inducing inflammatory response in human AVIC.

**Figure 1:**
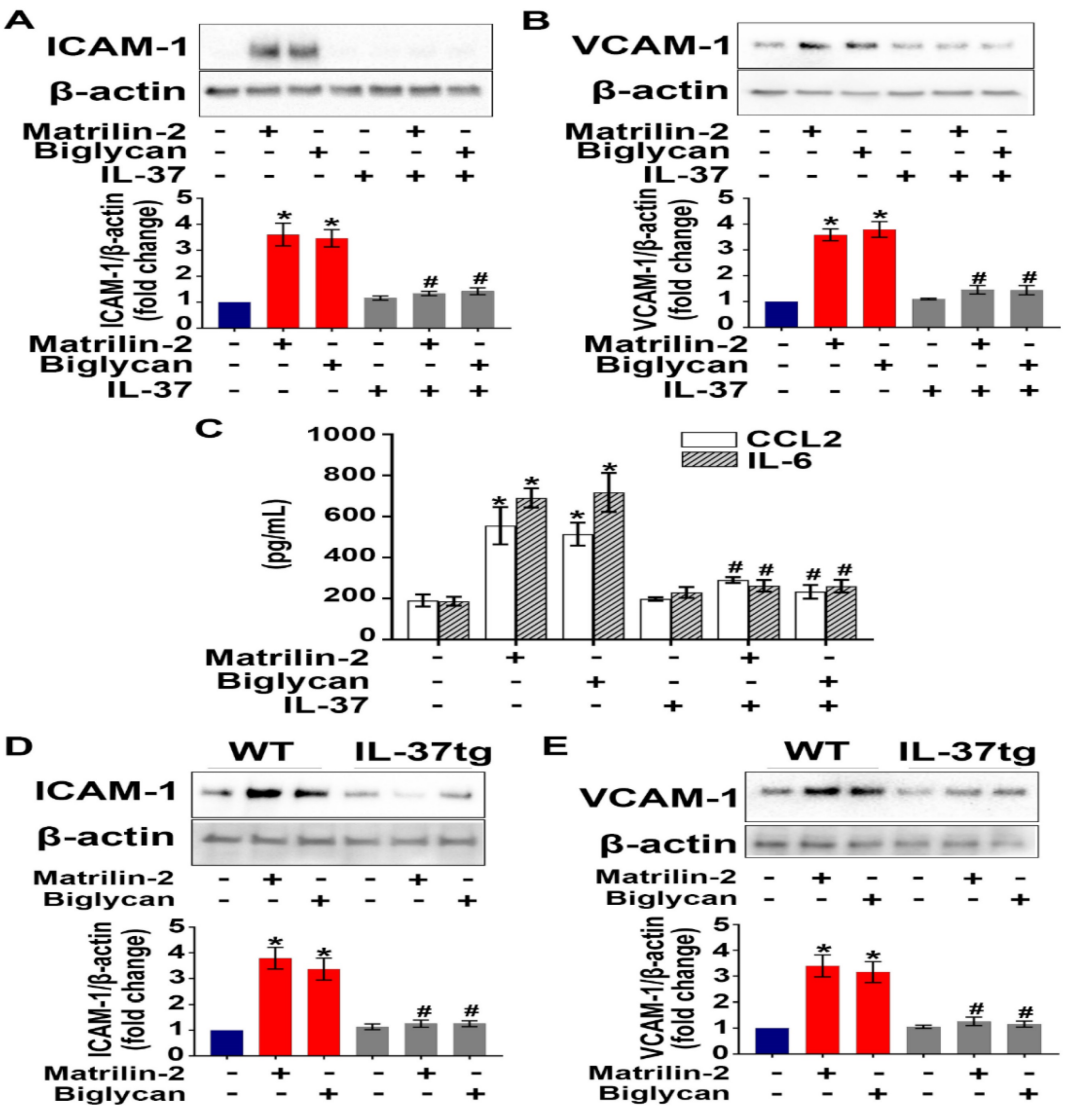
** Recombinant IL-37 suppresses soluble ECM proteins-induced adhesion molecules and pro-inflammatory cytokines in AVICs. (A** and** B)** Normal human AVICs were treated with recombinant IL-37 (1 ng/mL) for 1 hour or left untreated, followed by stimulation with recombinant matrilin-2 (2 µg/mL) or biglycan (0.2 µg/mL) for 48 hours. Representative immunoblots (*upper*) and densitometric data (*lower*) show that IL-37 abolished the effect of matrilin-2 and biglycan on the production of ICAM-1 and VCAM-1. Values are mean ± SEM, n = 4 donors. *P<0.05 vs. control. ^#^*P*<0.05 vs. matrilin-2 alone or biglycan alone. **(C)** Cytokine production of AVICs after stimulated with recombinant matrilin-2 (2 µg/mL) or biglycan (0.2 µg/mL) for 48 hours. IL-37 suppressed CCL2 and IL-6 production in AVIC exposed to soluble ECM proteins. Values are mean ± SEM, n = 3 donors. *P<0.05 vs. control. ^#^*P*<0.05 vs. matrilin-2 alone or biglycan alone. **(D** and** E)** AVICs from wild type mice and IL-37tg mice were treated with recombinant matrilin-2 (2 µg/mL) or biglycan (0.2 µg/mL) for 48 hours or left untreated. Representative immunoblots (*upper*) and densitometric data (*lower*) show that expression of IL-37 in murine AVICs abolished the effect soluble ECM proteins on the production of ICAM-1 and VCAM-1. Values are mean ± SEM, n = 4 mice per group. *P<0.05 vs. control. ^#^*P*<0.05 vs. matrilin-2 alone or biglycan alone.

To determine the effect of IL-37 on AVIC inflammatory response to soluble ECM proteins, we applied recombinant IL-37 (1 ng/mL) to AVICs prior to exposure to recombinant matrilin-2 or biglycan. As shown in **Figure 1A**-**C**, recombinant IL-37 suppressed the up-regulation of adhesion molecules (ICAM-1 and VCAM-1) and cytokines (CCL2 and IL-6) in AVICs exposed to either recombinant matrilin-2 or biglycan. WT mice do not express IL-37, while IL-37tg mice express this human cytokine 22, 31. Further, we treated AVICs from IL-37tg and WT mice with soluble ECM proteins matrilin-2 or biglycan. Immunoblotting data revealed that expression of IL-37 in AVICs abolished the production of ICAM-1 and VCAM-1 in response to soluble ECM proteins (**Figure 1D** and **1E**). These results demonstrate that IL-37 negatively regulates AVIC inflammatory response to soluble ECM proteins.

### Recombinant IL-37 inhibits PKR-*NF-*κ*B* activation induced by soluble ECM proteins

**Figure 2:**
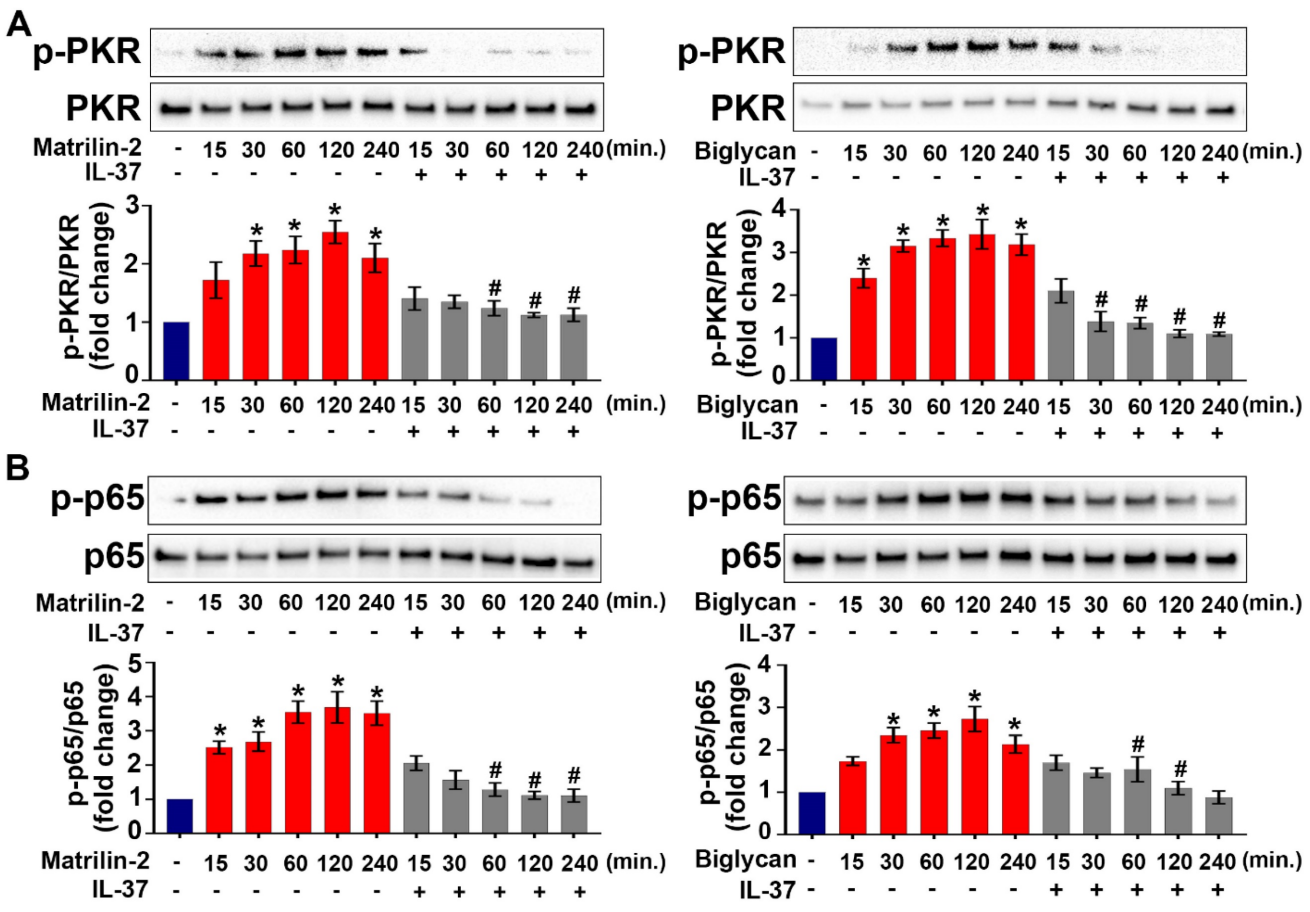
** IL-37 inhibits ECM protein-induced PKR and NF-κB activation. (A** and **B)** Normal human AVICs were treated with recombinant IL-37 (1 ng/mL) for 1 hour or left untreated. The cells were then stimulated with recombinant matrilin-2 (2 µg/mL) or biglycan (0.2 µg/mL) for different times. Representative immunoblots (*upper*) and densitometric data (*lower*) show that IL-37 markedly reduced the phosphorylation of PKR and NF-κB p65. Values are mean ± SEM, n = 4 donors. *P<0.05 vs. control. ^#^*P*<0.05 vs. cells treated with matrilin-2 alone or biglycan alone at the same time point.

Our recent work showed that the PKR-NF-κB signaling pathway plays a critical role in mediating AVIC inflammatory response to ECM proteins 11. To test the hypothesis that recombinant IL-37 suppresses the PKR-NF-κB signaling pathway in AVICs, we treated cells with recombinant IL-37 before exposure to recombinant matrilin-2 or biglycan. As shown in **Figure 2A** and **2B**, PKR and NF-κB were activated by soluble ECM proteins, and the activation was markedly reduced by the presence of recombinant IL-37. The results indicate that IL-37 suppresses the inflammatory response of AVICs to soluble ECM proteins via inhibition of the PKR-NF-κB signaling pathway.

### Neutralization of IL-18Rα only attenuates the anti-inflammatory effect of recombinant IL-37 on human AVICs

**Figure 3:**
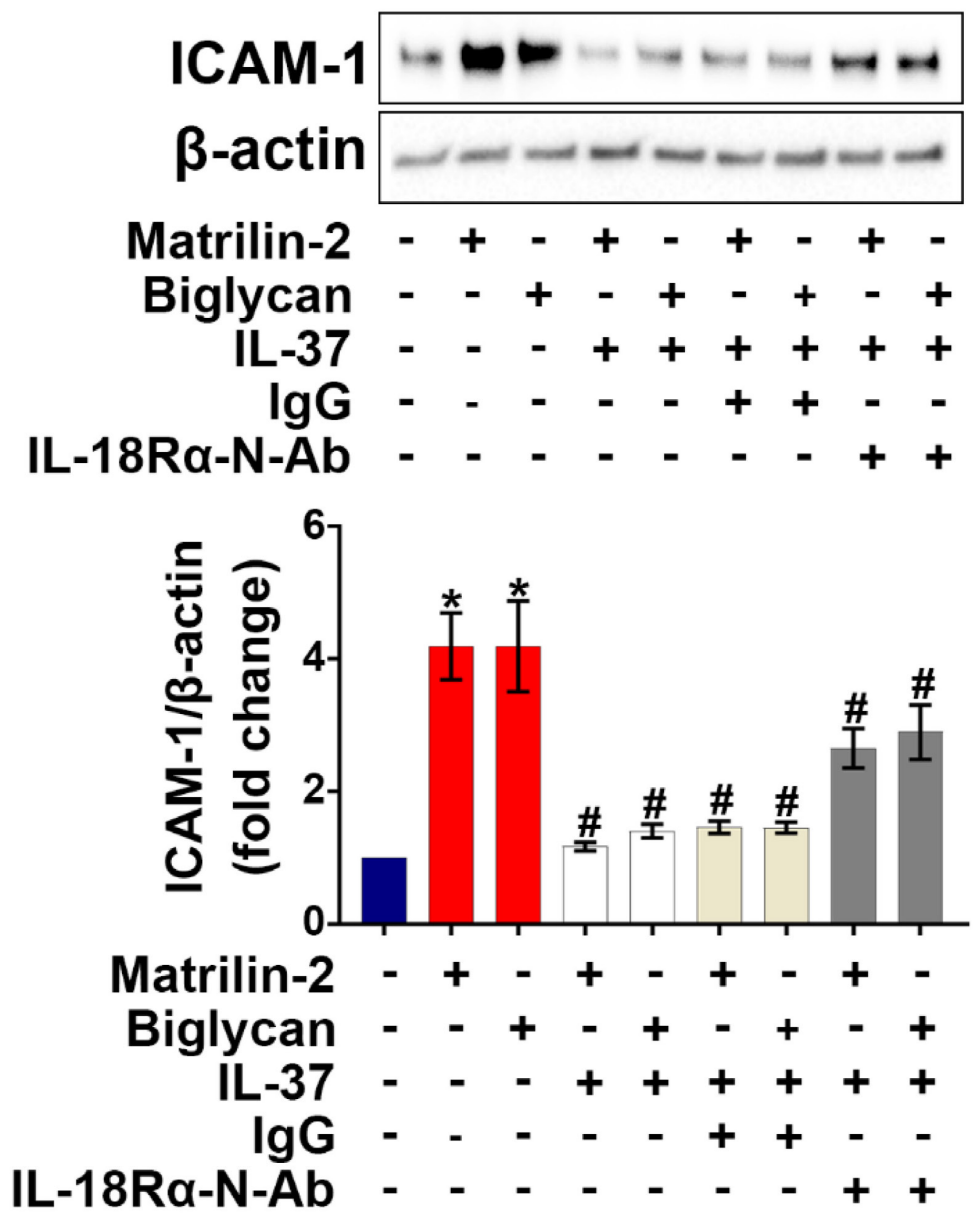
** Neutralization of IL-18Rα attenuates the anti-inflammatory effect of IL-37 on human AVICs.** Human AVICs were treated with IL-18Rα neutralizing antibodies (5 µg/mL) prior to recombinant IL-37. ICAM-1 levels were examined 48 hours after stimulation with recombinant matrilin-2 (2 µg/mL) or biglycan (0.2 µg/mL). Representative immunoblots (*upper*) and densitometric data (*lower*) show that neutralization of IL-18Rα attenuated the effect of IL-37 in suppressing ICAM-1 levels. Values are mean ± SEM, n = 4 donors. *P<0.05 vs. control. ^#^*P*<0.05 vs. cells treated with matrilin-2 alone or biglycan alone.

IL-18 is known to exert pro-inflammatory actions through binding to IL-18Rα 32, while previous studies have demonstrated that IL-37 utilizes IL-18Rα to exert its anti-inflammatory effects 33. We applied various dosages (1.5, 2.5, and 5 µg/mL) of IL-18Rα neutralizing antibodies to determine the optimal dose for blocking IL-18Rα activation on AVICs induced by pro-inflammatory stimulation*.* As shown in **Figure S2**, IL-18Rα neutralizing antibody at 5 µg/mL markedly reduced the expression of ICAM-1 in human AVICs exposed to IL-18. To determine whether IL-37 utilizes IL-18Rα to inhibit soluble ECM protein-induced AVIC inflammatory response, cells were pre-treated with IL-18Rα neutralizing antibody. Neutralization of IL-18Rα attenuated, but did not abolish, the anti-inflammatory effect of IL-37 on human AVICs exposed to soluble ECM protein (**Figure 3**). Our findings indicate that interaction of extracellular IL-37 with IL-18Rα is one of the mechanisms by which IL-37 inhibits soluble ECM protein-induced AVIC inflammatory response. Recombinant IL-37 can also utilize an IL-18Rα-independent mechanism to inhibit AVIC inflammatory response to soluble ECM proteins.

### Human AVICs internalize recombinant IL-37 in an IL-18Rα-independent fashion

**Figure 4:**
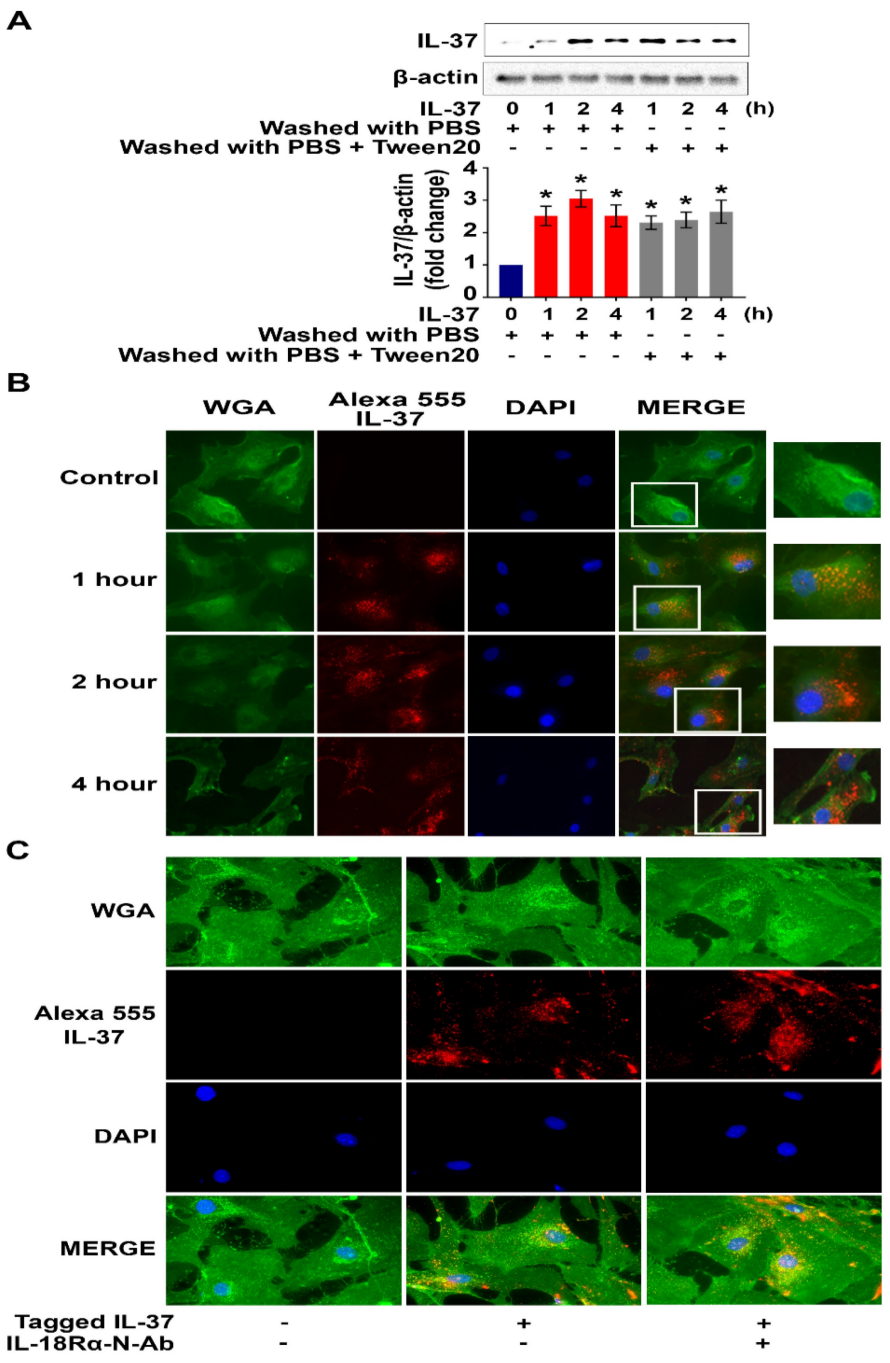
** Recombinant IL-37 internalized by human AVICs in an IL-18Rα-independent fashion. (A)** Human AVICs were left untreated or treated with recombinant IL-37 (1 ng/mL) for various times. After treatment, cells were washed with either PBS or PBS containing detergent. Representative immunoblots (*upper*) and densitometric data (*lower*) show that treatment with recombinant IL-37 increased cellular IL-37 levels. Values are mean ± SEM, n = 4 donors. *P<0.05 vs. control. **(B)** Human AVICs were treated with tagged IL-37 protein. Representative images of immunofluorescence staining show tagged IL-37 (red) is localized in the perinuclear region of human AVICs. Alexa Fluor 488 conjugate of wheat germ agglutinin (WGA) was applied to label plasma membrane (green). DAPI (40 ,6-diamidino-2-phenylindole) was used to stain nuclei (blue). Original magnification, 40x objective.** (C)** Representative immunofluorescence staining for tagged IL-37 (red), WGA (green), and DAPI (blue) on human AVICs. Original magnification, 63x objective. Neutralization of IL-18Rα does not alter the internalization of recombinant IL-37 into human AVICs.

To test the hypothesis that human AVICs internalize recombinant IL-37, cells were treated with recombinant IL-37 for 1 hour to 4 hours and washed with either PBS or PBS-containing detergent prior to harvest. Interestingly, we observed a marked increase in cellular levels of IL-37 (**Figure 4A**). To verify cellular uptake of recombinant IL-37, human AVICs were treated with recombinant IL-37 tagged with Alexa-555 (tagged IL-37). Microscopic observation confirmed that tagged IL-37 is present in the cytoplasm and in the perinuclear region (**Figure 4B**). To examine whether IL-18Rα is involved in the internalization of IL-37 into human AVICs, we treated cells with IL-18Rα neutralizing antibodies prior to incubation with tagged IL-37. As shown in **Figure 4C**, neutralization of IL-18Rα did not alter the internalization of tagged IL-37. Thus, human AVICs internalize recombinant IL-37 using IL-18Rα-independent mechanisms.

### Inhibition of endocytic pathways reduces the anti-inflammatory potency of recombinant IL-37 on human AVICs

**Figure 5:**
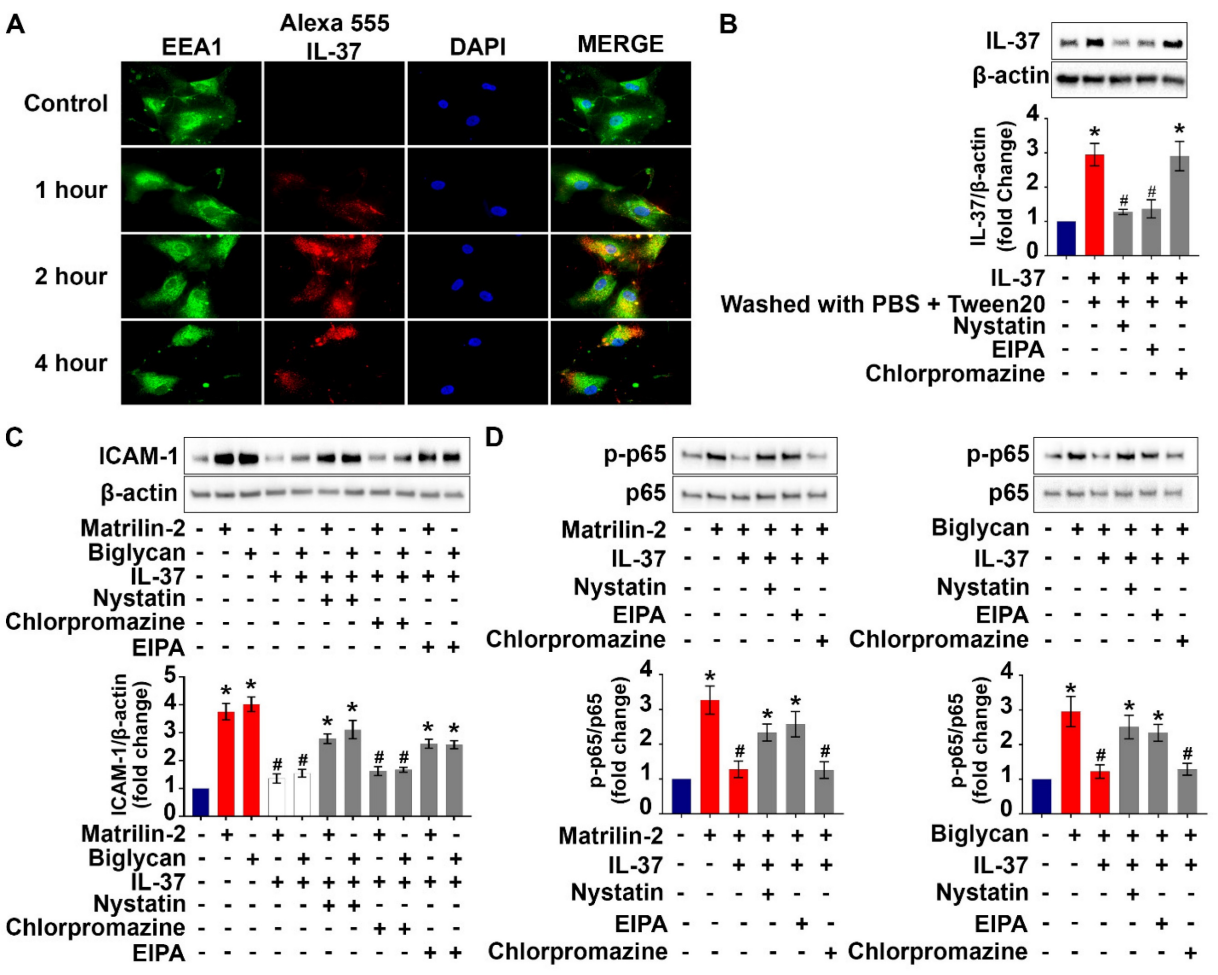
**Internalization of recombinant IL-37 into human AVICs is associated with early endosome and blocking the endocytosis pathway reduced the internalization and anti-inflammatory potency of recombinant IL-37. (A)** Human AVICs were treated with tagged IL-37 protein. Representative images of immunofluorescence staining show tagged IL-37 (red), EEA1 (green), and DAPI (blue). Original magnification, 63x objective. IL-37 is partially co-localized with EEA1 (early endosome). **(B)** Human AVICs were treated with specific inhibitors of endocytic pathways (Nystatin (10 µM), EIPA (20 µM) and Chlorpromazine (10 µM)) prior to recombinant IL-37 (1 ng/mL). Representative immunoblots (*upper*) and densitometric data (*lower*) show that Nystatin and EIPA reduce cellular levels of IL-37 in human AVICs treated with recombinant IL-37. Values are mean ± SEM, n = 4 donors. *P<0.05 vs. control. ^#^*P*<0.05 vs. cells treated with matrilin-2 alone or biglycan alone. **(C** and **D)** Human AVICs were treated with specific inhibitors of endocytic pathways prior to recombinant IL-37 (1 ng/mL) and matrilin-2 (2 µg/mL) or biglycan (0.2 µg/mL). Representative immunoblots (*upper*) and densitometric data (*lower*) show that inhibition of caveolae*-*mediated endocytosis or macropinocytosis attenuates the effect of recombinant IL-37 on inhibiting the activation of NF-κB, and the levels of ICAM-1 in human AVICs exposed to soluble ECM protein. Values are mean ± SEM, n = 4 donors. *P<0.05 vs. control. ^#^*P*<0.05 vs. cells treated with matrilin-2 alone or biglycan alone.

Cell surface proteins and some extracellular proteins could be internalized into cytoplasm through endocytic pathways 34. We performed co-localization analysis of labeled recombinant IL-37 and endosomes to understand if endocytosis is involved in the internalization of recombinant IL-37 by human AVICs. The results of immunofluorescence staining revealed that some tagged IL-37 is colocalized with early endosome (EEA1) after 2 hours of incubation (**Figure 5A**). In contrast, no colocalization of tagged IL-37 with late endosome (Rab7) was observed (**Figure S3**). The data indicate that early endosome is involved in internalization of recombinant IL-37.

To identify the endocytic pathway responsible for the internalization of recombinant IL-37 into human AVICs, we used pharmacological inhibitors of three distinct endocytic pathways (nystatin for the caveolin-endocytic pathway, chlorpromazine for clathrin-endocytic pathway, and amiloride-EIPA for macropinocytosis). Nystatin and amiloride inhibited the internalization of recombinant IL-37, resulting in a reduction of the inhibitory effects of recombinant IL-37 on NF-κB activation and downregulation of ICAM-1 expression (**Figure 5B-D**). However, these inhibitors did not attenuate the endocytosis-independent effects of recombinant IL-37 on PKR activation (**Figure S4**). Together, these results reveal that human AVICs are capable of internalizing recombinant IL-37 through endocytosis and that blocking the endocytosis pathway reduces the inhibitory effect of recombinant IL-37 on NF-κB activation in human AVICs. Our findings indicate that the effect of intracellular IL-37 on NF-κB is not through inhibition of PKR.

### Nuclear translocation is involved in the mechanism underlying the anti-inflammatory effect of intracellular IL-37

**Figure 6:**
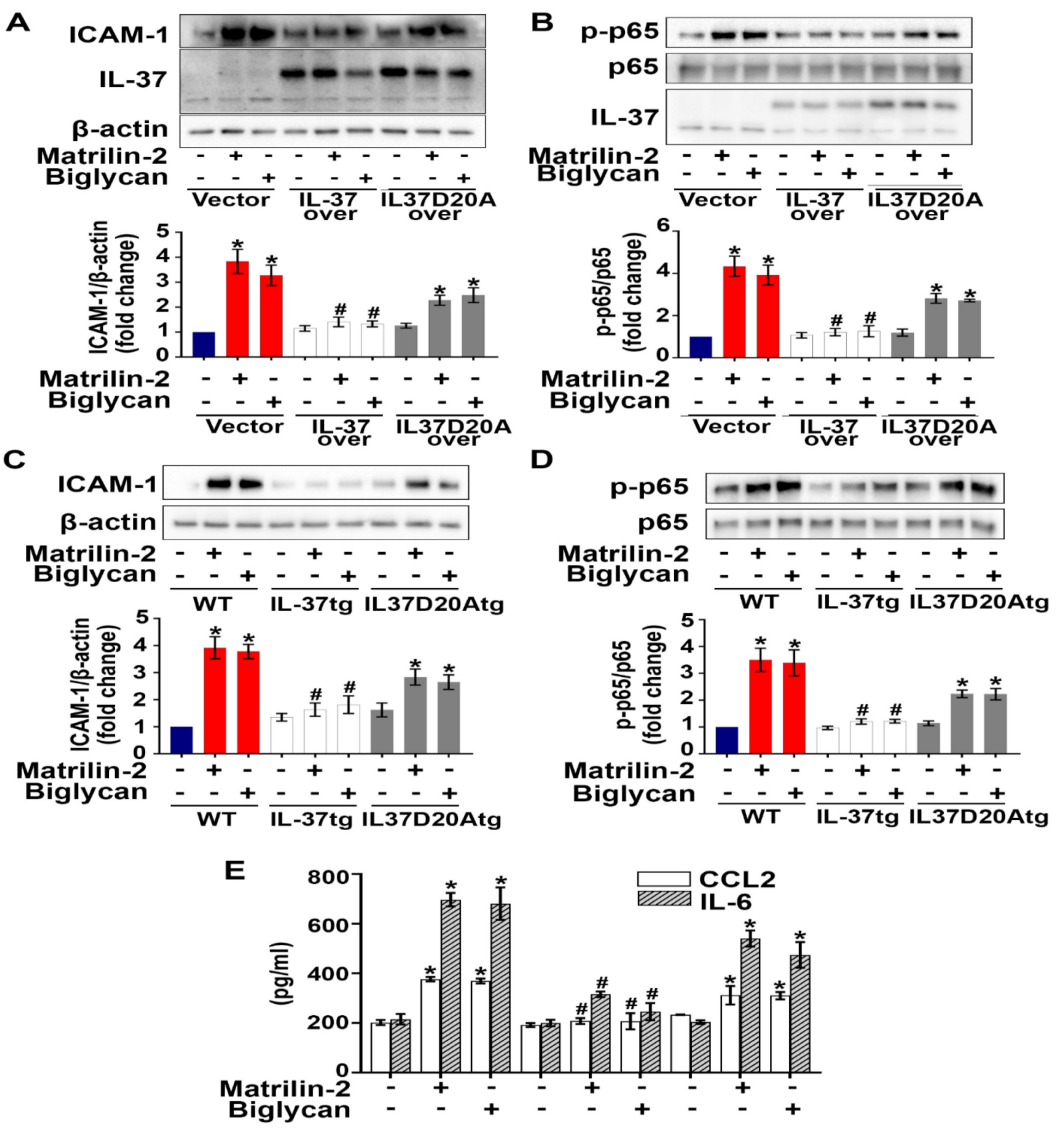
**Nuclear translocation appears to be involved in the mechanism underlying the anti-inflammatory effect of intracellular IL-37. (A** and **B)** Human AVICs were overexpressed with plasmid EGFP-IL-37 and EGFP-IL-37D20A and treated with matrilin-2 (2 µg/mL) or biglycan (0.2 µg/mL) for 48 hours. While expression of EGFP-IL-37 abolished NF-κB, and ICAM-1; expression EGFP-IL-37D20A did not. Values are mean ± SEM, n = 4 donors. *P<0.05 vs. control. ^#^*P*<0.05 vs. cells treated with matrilin-2 alone or biglycan alone. **(C-E)** AVICs from wild type, IL-37tg and IL-37D20Atg mice were treated with or without recombinant matrilin-2 (2 µg/mL) or biglycan (0.2 µg/mL). While expression of IL-37 in murine AVICs markedly reduced the phosphorylation of NF-κB p65 and inflammatory response, expression of mutant IL-37 (D20A that has no nucleus-targeting sequence) did not. Values are mean ± SEM, n = 4 mice per group. *P<0.05 vs. control. ^#^*P*<0.05 vs. cells treated with matrilin-2 alone or biglycan alone.

We hypothesized that the action site of intracellular IL-37 in AVICs is in the nucleus, as intracellular IL-37 translocates to the nucleus in LPS-stimulated RAW 264.7 macrophages 22, 26. To identify the action site of IL-37 in human AVICs, we exposed IL-37-overexpressing human AVICs to LPS for 2-8 hours prior to collection of nuclear and cytoplasmic fractions. As shown in **Figure S5**, IL-37 was detected in the nuclear fraction after exposure to LPS. However, this localization was not observed in human AVICs overexpressing IL-37D20A, an IL-37 mutant that lacks nuclear substituting an aspartic acid (D) to an alanine (A) at position 20 in IL-37b. To explore the mechanism by which intracellular IL-37 exerts its anti-inflammatory effect, we examined the effect of recombinant IL-37 on TLR4-mediated NF-κB activation. While TLR4 agonist LPS-induced IL-37 translocation into the nucleus in human AVICs with or without treatment with recombinant IL-37, pre-treatment with recombinant IL-37 for 24 hours increased IL-37 levels in both the cytoplasm and nucleus and reduced NF*-*κ*B* intranuclear translocation following TLR4 stimulation (**Figure S6**). Overexpression of IL-37 in human AVICs and in AVICs of IL-37tg mice suppressed NF-κB activation, as well as the expression of ICAM-1 and pro-inflammatory cytokines induced by soluble ECM proteins. In contrast, overexpression of IL-37D20A in both human and mouse AVICs had much attenuated anti-inflammatory effect (**Figure 6A-E**). Collectively, our data suggest that intracellular IL-37 primarily exerts its anti-inflammatory effect by targeting the nucleus.

## Discussion

CAVD is a chronic inflammatory disease 35. The pathobiology of this disease involves inflammatory responses of AVICs to soluble ECM proteins, with inflammation promoting fibrosis and calcification 8, 35, 36. Currently, there is no pharmacological intervention of CAVD progression 37. The present study provides evidence that: (1) recombinant IL-37 potently suppresses AVIC inflammatory response to soluble ECM proteins, (2) human AVICs are capable of internalizing recombinant IL-37 via the endocytic mechanism, and (3) intracellular IL-37 exerts its anti-inflammatory effect through a nucleus-targeting mechanism and by inhibition of the NF*-*κB signaling pathway. Our results demonstrate the potent anti-inflammatory effect of IL-37 on human AVICs and reveal that recombinant IL-37 exerts its anti-inflammatory effect by acting in both extracellular and intracellular compartments. The findings suggest that recombinant IL-37 has therapeutic potential for prevention of CAVD progression.

Chronic inflammation contributes to the development and progression of CAVD, with soluble ECM proteins acting as DAMPs. We previously reported that soluble ECM proteins up-regulate AVIC osteogenic and fibrogenic activities associated with the progression of CAVD through TLR-dependent mechanisms 8-11. Many studies demonstrate that IL-37 inhibits the production of IL-6 and TNF-α induced by LPS in monocytes and endothelial cells 38, 39. In addition, IL-37 reduces myocardial inflammation and improves cardiac function in old endotoxemic mice 38. Patients with CAVD have lower levels of IL-37 in their aortic valve 4. Recombinant IL-37 has been shown to suppress AVIC inflammatory response, as well as valvular thickening induced by TLR2/4 agonists 7, 27. Our data in the present study demonstrate that IL-37 suppresses the production of inflammatory mediators in human and murine AVICs exposed to soluble ECM proteins. Cell adhesion molecules (ICAM-1 and VCAM-1) and cytokines (CCL2 and IL-6) have been implicated in the pathogenesis of chronic inflammatory diseases, including CAVD 40. The ability of IL-37 to suppress the inflammatory activities in AVICs induced by endogenous stimulants underscores its potential for suppression of valvular inflammation and for prevention of CAVD progression.

IL-37 has been reported to exert its anti-inflammatory effect by interacting with IL-18Rα 4, 41. However, it is unclear whether IL-18Rα has a major role in mediating the anti-inflammatory function of IL-37 in AVICs exposed to soluble ECM proteins. Our results in the present study show that neutralizing IL-18Rα only partially reduces IL-37's ability to suppress the inflammatory response of AVICs exposed to soluble ECM proteins. This finding suggests that IL-37 may employ other mechanisms, independent of IL-18Rα, to inhibit the inflammatory response of AVICs to soluble ECM proteins. Recent studies have reported an intracellular anti-inflammatory mechanism of endogenous IL-37 31, 41. Our study provides evidence demonstrating that recombinant IL-37 exerts its anti-inflammatory action on human AVICs through both extracellular and intracellular mechanisms. It is interesting that neutralization of IL-18Rα does not affect the internalization of tagged IL-37. This observation suggests two possibilities. One is that human AVICs employ IL-18Rα-independent mechanism(s) for the internalization of recombinant IL-37. Indeed, proteins can undergo internalization into cells through receptor-independent mechanisms, including pinocytosis. Alternatively, the IL-18Rα-neutralizing antibody may inadvertently facilitate cellular internalization of the antibody-antigen-ligand complex (mAb/IL-18Rα/IL-37) by the Fc-receptor-mediated endocytosis. Nevertheless, our results show that treatment with neutralizing antibody against IL-18Rα only moderately reduces IL-37's ability to suppress the inflammatory response of AVICs to soluble ECM proteins. In review of the inhibitory effect of this antibody on IL-37 by blocking IL-18Rα signaling, it is reasonable to speculate that the mAb/IL-18Rα/IL-37 complex, if formed, may not significantly attenuate the anti-inflammatory effect of internalized recombinant IL-37.

Cells can uptake extracellular fluids, plasma membrane proteins, ligands and lipids through endocytosis. This process has several pathways, including clathrin-mediated endocytosis, caveolae-mediated endocytosis, and macropinocytosis 42, 43. Annexin A2 is an example that protein exerts its anti-inflammatory effect after internalization via endocytosis 44. The findings of the present study reveal that the internalization of recombinant IL-37 into human AVICs is facilitated by caveolin-mediated endocytosis and macropinocytosis, and that recombinant IL-37 is associated with early endosomes. Interestingly, inhibition of these endocytic pathways resulted in a reduction of IL-37 internalization and its overall anti-inflammatory effect in AVICs. The present study provides insight into the mechanism by which recombinant IL-37 exerts its anti-inflammatory action on human AVICs and may facilitate the development of therapeutic approaches for prevention of CAVD progression.

IL-37D20A is a mutated form of IL-37 in which alanine replaces aspartic acid at position 20, making it resistant to cleavage by caspase-1 and unable to translocate to the nucleus 25. Cytoplasmic*-*nuclear trafficking of IL-37 was noted in LPS-stimulated human AVICs overexpressing IL-37, but not in cells overexpressing IL-37D20A. Previous studies have reported that macrophages from IL-37D20A transgenic mice are unable to suppress cytokine production induced by LPS 31. In the present study, we observed that overexpression of IL-37 in both human and mouse AVICs effectively reduced the levels of ICAM-1, CCL2 and IL-6 following being exposed to soluble ECM proteins, while IL-37D20A had a much weaker effect. It is known that corticosteroids translocate into the nucleus and bind to specific cis-acting DNA sequences 45. Internalized/intracellular IL-37 appears to utilize a similar mechanism to suppress AVIC inflammatory response as it translocates to the nucleus and inhibit pro-inflammatory transcription factors 46. Thus, internalized recombinant IL-37 appears to act similarly as endogenous intracellular IL-37.

PKR is a ubiquitously expressed serine and threonine protein kinase 47. PKR has been shown to activate NF-κB to induce inflammatory response in a variety of cell types 48, 49. The inflammatory response induced by soluble ECM proteins in human AVICs involves PKR/NF-κB signaling 11. Interestingly, treatment with recombinant IL-37 significantly reduces the activation of PKR and NF-κB upon exposure to soluble ECM proteins. Inhibition of the PKR/NF-κB pathway maybe an important mechanism by which IL-37 suppresses AVIC inflammatory response to soluble ECM proteins. However, IL-37 may also suppress other inflammatory signaling pathways. A study reported that IL-37 inhibits the proliferation and invasion of human cervical cancer cells by suppressing signal transducer and activator of transcription (STAT)3 50. Further studies are needed to evaluate the effect of IL-37 on other pro-inflammatory pathways in human AVICs. Intracellular IL-37 can bind to Smad3 and subsequently translocate to the nucleus 31. Our findings show that LPS induces IL-37 intranuclear translocation. While NF-κB translocates to the nuclei from the cytoplasm in response to LPS stimulation, pre-treatment of AVICs with recombinant IL-37 increases the level of IL-37 in the nuclei following LPS stimulation and attenuates NF-κB intranuclear translocation. These findings imply that internalized/intracellular IL-37 inhibits the inflammatory response by two actions: preventing NF-κB intranuclear translocation and inhibiting NF-κB activity in the nucleus. Further studies are needed to elucidate the specific mechanism and the interaction between IL-37 and NF-κB.

Overall, our study highlights the novel finding that recombinant IL-37 exerts a potent anti-inflammatory effect on human AVICs through both intracellular and extracellular actions. As a recombinant cytokine, IL-37 is druggable. Its potent anti-inflammatory effect and unique mechanism of action make it promising for the prevention of CAVD progression and for the treatment of other chronic inflammatory diseases.

## Conclusion

The present study highlights the novel findings that human AVICs can uptake recombinant IL-37 via endocytosis and that intracellular IL-37 exerts its anti-inflammatory effect in aortic valve cells via nucleus-targeting mechanism. These findings support the notion that recombinant IL-37 could be developed to a pharmacological intervention for prevention of CAVD progression by suppression of valvular inflammation.

## Supplementary Material

Supplementary figures.

## Abbreviations

AVICs: aortic valve interstitial cells
CCL2: C-C motif chemokine ligand 2
CAVD: calcific aortic valve disease
DAMPs: damage-associated molecular patterns
DAPI: diamidino-2-phenylindole
ECM: extracellular matrix
EEA: early endosome antigen
EGFP: enhanced green fluorescent protein
FBS: fetal bovine serum
ICAM-1: intercellular adhesion molecule 1
IL: interleukin
IL-18Rα: IL-18 receptor α-chain
IL-37tg: IL-37 transgenic
IL-37D20A: mutated D to A site in IL-37b
LPS: lipopolysaccharide
NF-κB: nuclear factor kappa-light-chain-enhancer of activated B cells
PBS: phosphate-buffered saline
PKR: double-stranded RNA-dependent protein kinase
Rab: Ras-related protein
STAT: signal transducer and activator of transcription
TLR: Toll-like receptors
TNF: tumor necrosis factor
VCAM-1: vascular cell adhesion protein 1
WGA: wheat germ agglutinin
WT: wild type
