# Supplementary material for: Recombinant IL-37 Exerts an Anti-inflammatory Effect on Human Aortic Valve Interstitial Cells through Extracellular and Intracellular Actions
Source: Int J Biol Sci. 2023 Jul 31;19(12):3908–19. doi: 10.7150/ijbs.85745 (PMC10411472; doi:10.7150/ijbs.85745)
Supplement: Supplementary file 1 — Supplementary figures. [file ijbsv19p3908s1.pdf]

Supplementary Figures

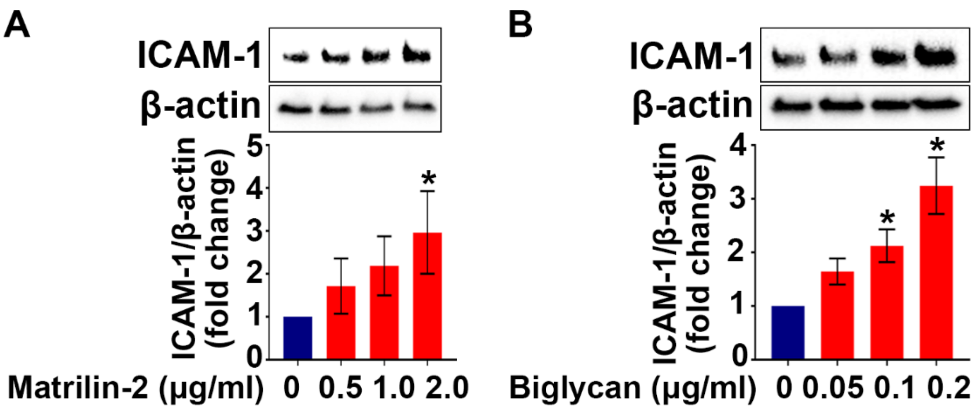

Figure S1. **Soluble ECM proteins induce AVICs inflammatory response.** Human AVICs were stimulated with recombinant matrilin-2 or biglycan with different concentrations. Values are mean ± SEM, n = 3 donors. *P<0.05 vs. control.

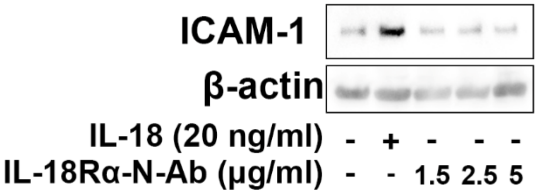

Figure S2. **Neutralization of IL-18Rα suppresses the upregulation of ICAM-1 expression induced by IL-18.** Human AVICs were stimulated with IL-18Rα neutralizing antibodies prior to recombinant IL-18 treatment. Neutralization of IL-18Rα markedly reduced ICAM-1 level in human AVICs following IL-18 treatment. n = 2 donors.

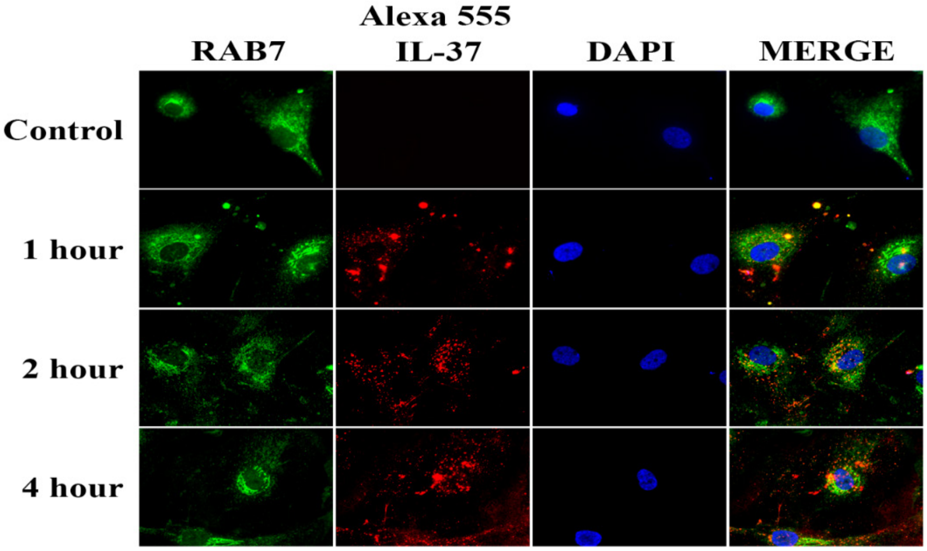

Figure S3. The internalization of recombinant IL-37 into human AVICs is associated with late endosomes. Cells were treated with tagged IL-37 protein. Representative images of immunofluorescence staining show that IL-37 did not colocalize with Rab7 (late endosomes). Red: Tagged IL-37; Green: Rab7; and Blue: DAPI. Original magnification, 63x objective.

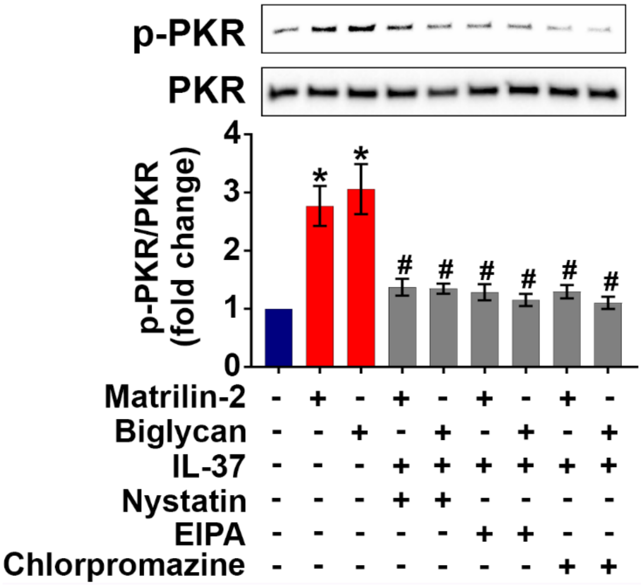

Figure S4. Endocytosis inhibitors do not attenuate the inhibitory effects of IL-37 on PKR phosphorylation. Human AVICs were treated with pharmacological inhibitors of endocytosis prior to recombinant IL-37 and matrilin-2 or biglycan treatment. n = 4 donors. *P<0.05 vs. control. $^{\#}P$<0.05 vs. cells treated with matrilin-2 alone or biglycan alone.

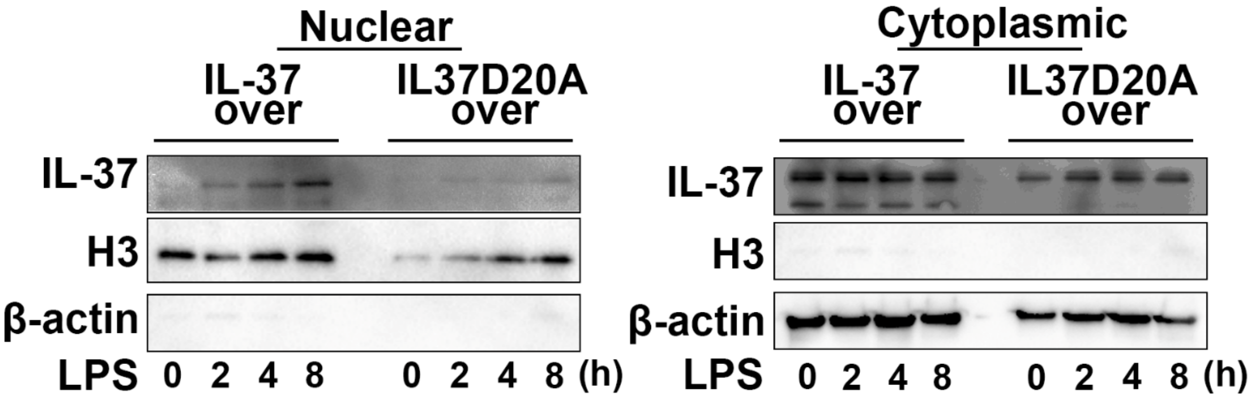

Figure S5. IL-37 expression is detected in nuclear fraction of AVICs overexpressing IL-37 but not in IL-37D20A. Nuclear and cytoplasmic fractions from human AVICs were extracted after cells overexpressed with plasmid EGFP-IL-37 and EGFP-IL-37D20A and treated with LPS (200 ng/mL). n = 2 donors.

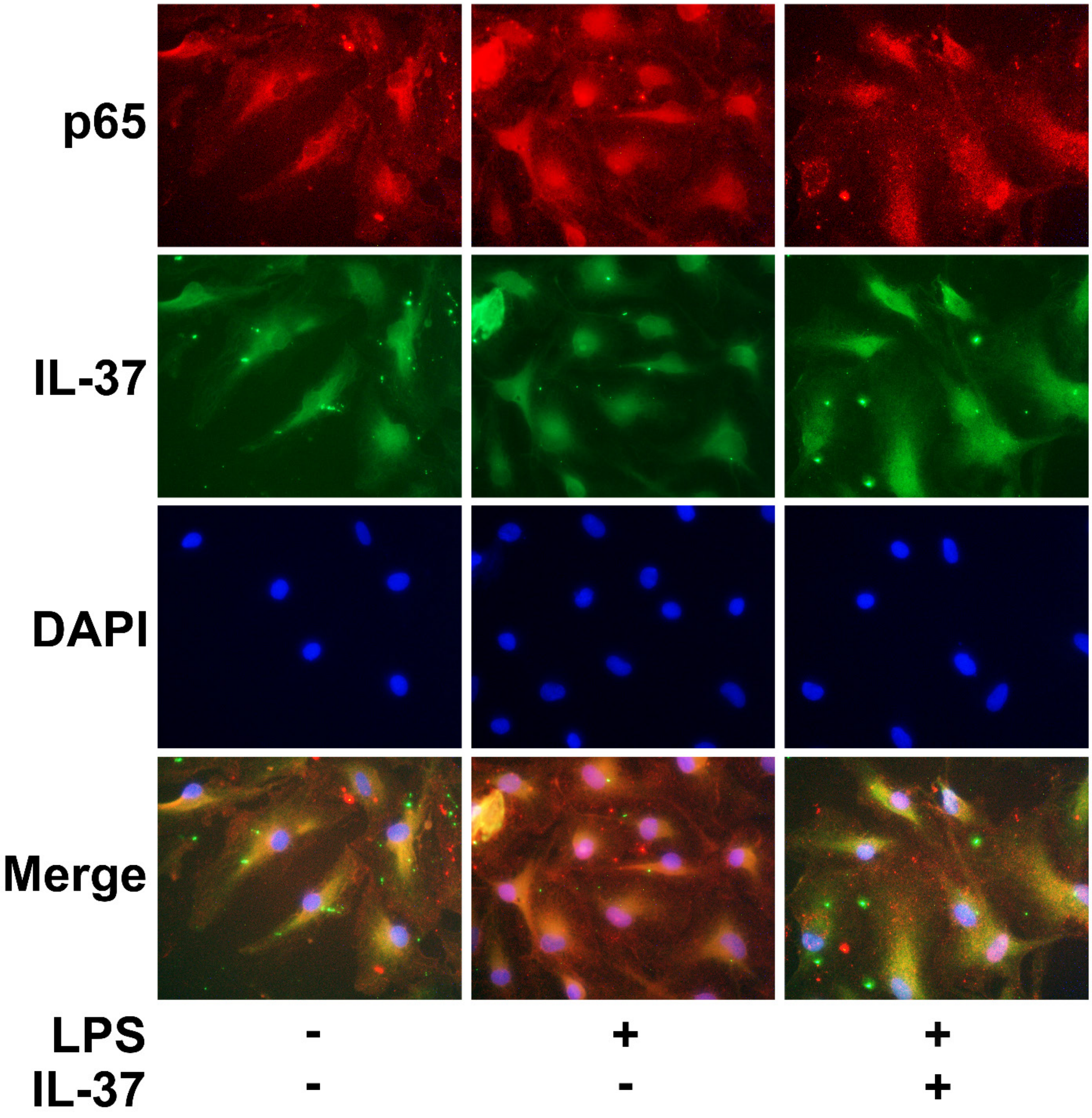

Figure S6. Treatment with recombinant IL-37 reduces NF-κB intranuclear translocation. Human AVICs were pre-treated with recombinant IL-37 at a concentration of 1 ng/mL for 24 hours followed by an exposure to Toll-like receptor 4 agonist LPS (200 ng/mL) for 4 hours. Representative images show that IL-37 translocates to the nucleus in response to LPS stimulation in cells with or without recombinant IL-37. Treatment with recombinant IL-37 increases nuclear level of IL-37 and attenuates NF-κB p65 intranuclear translocation in cells exposed to LPS. Original magnification, 40x objective.
